# Crossing Wallace’s line: an evolutionarily young gibbon ape leukemia virus like endogenous retrovirus identified from the Philippine flying lemur (*Cynocephalus volans*)

**DOI:** 10.1038/s41598-025-94582-1

**Published:** 2025-03-21

**Authors:** Kyriakos Tsangaras, Jens Mayer, Alex D. Greenwood

**Affiliations:** 1 Department of Life and Health Sciences, University of Nicosia, Nicosia, Cyprus; 2 Institute of Human Genetics, Medical Faculty, University of Saarland, Homburg, Germany; 3 Department of Wildlife Diseases, Leibniz Institute for Zoo and Wildlife Research (IZW), Berlin, Germany; 4 Department of Cardiovascular Genetics and the Laboratory of Forensic Genetics, Cyprus Institute of Neurology and Genetics, Nicosia, Cyprus; 5 School of Veterinary Medicine, Freie Universität Berlin, Berlin, Germany

**Keywords:** Wallace’s line, Endogenous retrovirus, Gammaretrovirus, Gibbon ape leukemia virus, Flying lemurs, Southeast Asian mammals, Evolution, Genomics

## Abstract

Wallace’s line is a biogeographical barrier to faunal movements between Southeast Asia and the Australo-Papuan region. There are exceptions among rodents and bats, few of which have crossed Wallace’s line. The gibbon ape leukemia viruses (GALV) and koala retrovirus (KoRV) have only been identified in wildlife on the Australo-Papuan side of Wallaces’s Line with the potential exception of partial sequences identified in two microbat fecal samples from China and a recently described GALV relative in a rodent from Africa. Here we describe a group of GALV-like endogenous retroviral sequences from the Southeast Asian flying lemur (*Cynocephalus volans*) representing the first known description of a primate relative which has been infected, and the germline colonized, by GALVs on the Southeast Asian side of Wallace’s Line.

## Introduction

Retroviruses are a diverse group of single stranded RNA viruses which convert their genomes to double-stranded DNA by reverse transcription and subsequently integrate the reverse transcribed DNA into the host genome^1,2^. The resulting provirus has all the necessary sequences to produce infectious virions^3^. If integration occurs in the germline, vertical transmission from parent to offspring can occur. Retroviruses that have infected the germline and are inherited in a Mendelian fashion are called Endogenous Retroviruses (ERVs)^1,2^. ERVs are found in all vertebrate genomes examined to date^4,5^ and have been associated with various diseases, e.g. neoplasia, autoimmune diseases and neurodegenerative diseases^6–8^.

Most ERVs are remnants of ancient exogenous retroviral infections that occurred millions of years ago^9–11^. The koala retrovirus (KoRV) and the complete Melomys Woolly Monkey Virus (cMWMV) are the only retroviruses currently known to be in the early process of colonizing their wildlife host germlines in the koala (*Phascolarctos cinereus*) and the rodent *Melomys leucogaster*, respectively^12,13^. Phylogenetically, the closest relatives of KoRV and cMWMV are the Gibbon ape leukemia viruses (GALV), a group of exogenous infectious retroviruses originally isolated exclusively from captive Gibbons in Thailand and a woolly monkey likely infected by a gibbon (WMV)^5,14^. GALV has not been detected in wild gibbons and has not been isolated from captive gibbons since 1979^15,16^. Lack of presence of the virus in the wild population suggests cross-species transmission in captivity from an unknown virus and from an unidentified host^17,18^. Recently it has been suggested that the infection of gibbons with GALV and woolly monkeys with WMV represent laboratory outbreaks that were exacerbated by human activities^17^.

The biogeographical and ecological connection between GALV in gibbons of SE Asia and KoRV in koalas in Australia remains obscure. The fauna of Southeast Asia, the Australo-Papuan region (Australia, New Guinea), and Wallacea (including the Philippines apart from Palawan) represent historic biogeographical realms that have limited natural faunal dispersion among them as demarcated by the Wallace, Huxley, and Lydekker Lines^19^. On the Australo-Papua side of the Wallace’s Line, endogenous retroviral sequences closely related to GALV have been described from the Australian rodent *Melomys burtoni* (MbRV), *Melomys burtoni* subspecies (MelWMV) from Wallacea in Indonesia, and *Melomys leucogaster* (cMWMV) from Papua New Guinea^13,14,18^. GALV-like retroviruses (FFRV1, HPG) were also identified from the Australian black flying fox, *Pteropus alecto*, a species that has habitat overlap with both gibbons and koalas, from the long-tongued nectar bat (*Macroglossus minimus*; MmGRV), and from the common blossom bat (*Syconycteris australis*; SaGRV). Two Yinpterochiropteran microbats from China were positive for GALV-like viruses (HIGRV, RhGRV), and are the only free-living wild Asian animals identified to date with sequences similar to GALV that have crossed Wallace’s Line^2,5^. An additional GALV-like sequence has been described in an African rodent suggesting the distribution of this viral group may be broader than expected^20^.

Despite the recent identification of several sequences related to KoRV and GALV, only a small fraction of mammalian genomes from the biodiverse geographic region adjacent to Wallace’s Line have been investigated. This lack of broad screening makes it difficult to draw conclusions with respect to the origin, distribution, and endogenization of this important GALV retroviral clade which includes two of the known actively endogenizing retroviruses. We therefore screened 31 publicly available genome assemblies of Southeast Asian mammals for KoRV and GALV-like retroviral sequences (Table S1). Our genome sequence screens identified ERVs closely related to Woolly Monkey Virus in the Philippine Flying Lemur (*Cynocephalus volans*). The ERVs represent the first GALV and KoRV-like sequences identified in a free-living mammal on the Southeast Asian side of Wallace’s Line. The results demonstrate that the transmission of GALV-like retroviruses are not entirely restricted to the Australo-Papuan side of Wallace’s Line. Moreover, we present here the first evidence of a GALV-like sequence in wild primate relatives. The identification of CynVolERV may serve as potential link between rodents and primates, offering new insights into the evolutionary history of these retroviruses.

## Results

### KoRV and GALV retrovirus screening in Southeast Asia mammalian genome sequences

**Fig. 1:**
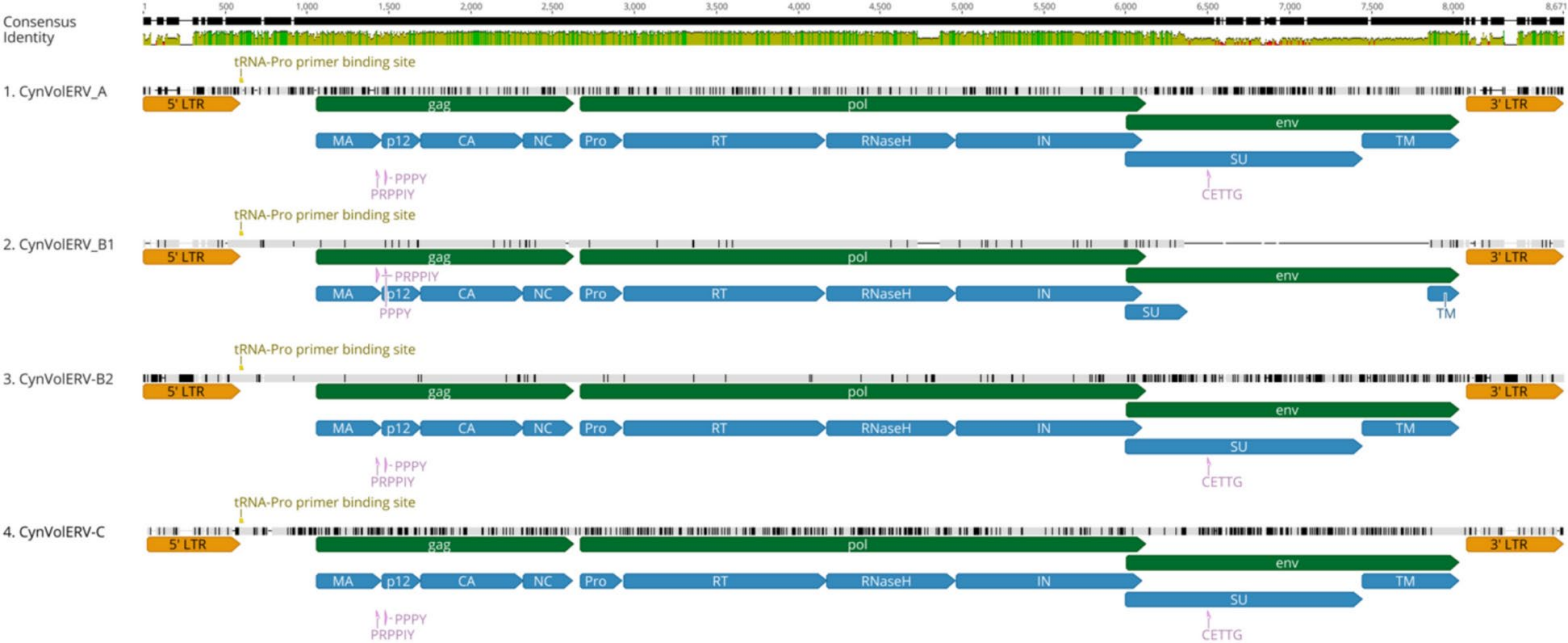
Multiple alignment of CynVolERV-A, CynVolERV-B1, CynVolERV-B2, and CynVolERV-C consensus sequences generated from proviruses extracted from the *Cynocephalus volans* genome sequence. Nucleotide positions identical to the consensus of all four sequences are indicated in light gray, mismatches are shown in black, gaps in the sequence alignment are shown as horizontal lines. Green, yellow and red bars above the alignment indicate the percent identity among the sequences (green, highest identity; red, lowest identity). Locations of proviral genes (*gag*, *pol*, *env*) and protein domains are indicated in green and blue. The following structural regions are shown: 5’ and 3’ long terminal repeats (LTRs) indicated in orange, primer binding site (PBS) (yellow), Cys-His box (grey), other retroviral motifs (pink), and the polypurine tract (PPT) (grey). Protein domain abbreviations: MA, matrix; p12, CA, capsid; NC, nucleocapsid; Pro, protease; RT, reverse transcriptase; RNase H, IN, integrase; SU, surface subunit; TM, transmembrane subunit.

KoRV (Genbank accession number KF786285) and GALV (KT724047) sequences obtained from NCBI were used as seed sequences for BLAST searches, using the megablast algorithm, in 31 different southeast (SE) Asia mammalian genome sequence assemblies obtained from NCBI Datasets^21^ (Table S1). Megablast searches produced multiple hits with pairwise identity higher than 85% for the Philippine Flying Lemur (*Cynocephalus volans*) genome exclusively. The rest of the mammalian genomes screened only produced a minimal number of hits that represented more distant ERV sequences. Megablast-positive hits plus 10,000 bp of up and downstream flanking genome sequence were extracted from the *C. volans* genome sequence. Extracted genome regions were screened using RepeatMasker^22^ to assess presence of endogenous retrovirus like sequences. Repeatmasker identified gammaretroviral sequences present in the extracted regions. We furthermore utilized RetroTector^23^ to reveal the presence of 434 proviral sequences and identified the boundaries of gammaretroviral genes and LTRs. Identified proviral sequences were multiply aligned using MAFFT^24^, with the resulting alignment revealing three distinct groups of KoRV/GALV-like retroviral sequences consisting of 86, 341, and 5 proviruses each. The second group of 341 sequences could be further subdivided into 2 subgroups based on presence or absence of a deletion within the *env* gene. The identified proviral sequence groups were termed *Cynocephalus volans* endogenous retroviruses CynVolERV-A, B1, B2, and C. A majority-rule based consensus was generated for each alignment with pairwise identities compared to KoRV and GALV reference sequences ranging between 62–86% (Fig. 1, Table S19). While all four proviral consensuses have *gag*, *pro*, *pol,* and *env* genes the CynVolERV-B1 consensus has a 133 bp deletion within the RNaseH region of *pol* that creates a downstream stop codon, as well as a 1487 nucleotide deletion in *env* (Fig. 1). NCBI Conserved Domain searches^25^ were also performed on the generated CynVolERV consensus sequences verifying RetroTector results.

### Analysis of CynVolERV proviruses

Our analysis of the *Cynocephalus volans* genome sequence identified 86 CynVolERV-A, 183 CynVolERV-B1, 158 CynVolERV-B2, and 5 CynVolERV-C proviral sequences (Fig. S1, Table S2). Phylogenetic analysis of the *Cynocephalus volans* proviral sequences confirmed the presence of the different subgroups identified (Fig. S2). Eighty three out of the 86 CynVolERV-A proviruses had *gag*, *pro*, *pol*, and *env* genes, and 73 out of 86 proviruses had full-length ORFs for those genes. Eight CynVolERV-A proviruses had either a gene missing partially or entirely and another five CynVolERV-A proviruses had a stop codon in one of the coding regions. We also determined the presence of retroviral motifs that are important for infectivity, specifically amino acid sequence motifs PRPPIY and PPPY located in the *gag* gene, and motif CETTG within the *env* gene. PRPPIY and PPPY motifs are known to play a role in viral release from the cell membrane, disruption of those motifs reduces the viral titer and viral budding^14^. The CETTG motif is present in infectious exogenous gammaretroviruses but often altered in ERVs^26^. The CETTG motif disruption reduces cytopathology^27^. All the CynVolERV-A proviruses had an intact PRPPIY motif, 84 proviruses had an intact PPPY motif, and 75 proviruses had an intact CETTG motif.

All the CynVolERV-B1 subgroup proviral sequences had *gag*, *pro*, *pol, and env* genes present. However, a 133 nt deletion in *pol* was found in 131 of the proviral sequences, and 179 out of 182 proviral sequences had a 1487 nt deletion within *env*. CynVolERV-B2 subgroup proviruses had a *gag* gene in 157 out of 158 proviral sequences. The *pol* gene region was present in all proviral sequences, but 21 of the proviral sequences harbored a 2216 nt deletion within the *pol* region. The *env* gene region was likewise present in all but 18 proviral sequences that harbored deletions of variable length within the *env* gene. Motif analysis for the CynVolERV-B group of viruses revealed the presence of a PQPPVL motif, instead of PRPPIY, in all the sequences except for one CynVolERV-B2 proviral sequence that had the PRPPIY motif. The PPPY motif was present in 35 CynVolERV-B1, and in 111 CynVolERV-B2 proviral sequences, while the rest of the proviral sequences had a TPPY motif instead. The CETTG motif in the *env* gene was absent in all CynVolERV-B1 proviruses, as the majority of the *env* coding region is missing, however a CETTG motif was present in all CynVolERV-B2 proviral sequences.

Analysis of CynVolERV-C proviral sequences verified the presence of all retroviral genes. Open reading frame analysis of the five CynVolERV-C proviral sequences indicated that one of the proviral sequences had a stop codon in the *gag* gene, while the proviruses appear to have protein coding potential for all the other retroviral genes. Motif analysis of the CynVolERV-C proviral sequences revealed presence of PQPPVL and TPPY motifs, instead of PRPPIY and PPPY, in *gag*, while the CETTG motif in *env* was found in all the sequences.

Important in this context, the presence of full-length ORFs and intact infectivity-related retroviral motifs indicate that the majority of CynVolERV-A proviruses could still potentially produce viral particles and be infectious. On the other hand, the majority of CynVolERV-B proviruses do not appear able to produce full-length retroviral proteins due to stop codons in their respective reading frames. Specifically, only nine identified CynVolERV-B2 proviruses could potentially produce viral particles. Four out of five CynVolERV-C proviral sequences identified could potentially produce viral particles.

We further investigated whether CynVolERV proviruses are transcribed. We analyzed a paired-end RNA-seq dataset (run accession number SRR27761458; study accession number PRJNA516733) previously generated from heart tissue of a *Cynocephalus volans* individual. We employed RNA STAR^28^ at Galaxy (https://usegalaxy.eu)^29^ to align reads from dataset SRR27761458 to CynVolERV-A, CynVolERV-B1, CynVolERV-B2, or CynVolERV-C proviral consensus sequences. Out of approx. 134 million read pairs, approx. 22,703 (0.02%) of reads aligned with CynVolERV-A, 120,373 (0.09%) of reads aligned with CynVolERV-B1, 118,934 (0.09%) of reads aligned with CynVolERV-B2, and 32,169 (0.02%) of reads aligned with CynVolERV-C. Therefore, transcriptome data indicated that proviruses of all four CynVolERV subgroups are expressed in heart tissue of *Cynocephalus volans*, and likely are expressed in other *C. volans* tissues as well. We note that our analysis did not identify individual CynVolERV proviruses as transcribed because of the overall high sequence similarities between CynVolERV proviruses, that makes unambiguous assignment of RNA-seq reads to specific proviruses difficult, if not impossible. Future studies will be needed to determine how many CynVolERV proviruses are transcriptionally active.

### CynVolERVs long terminal repeat analysis indicates numbers of LTR variants

**Fig. 2:**
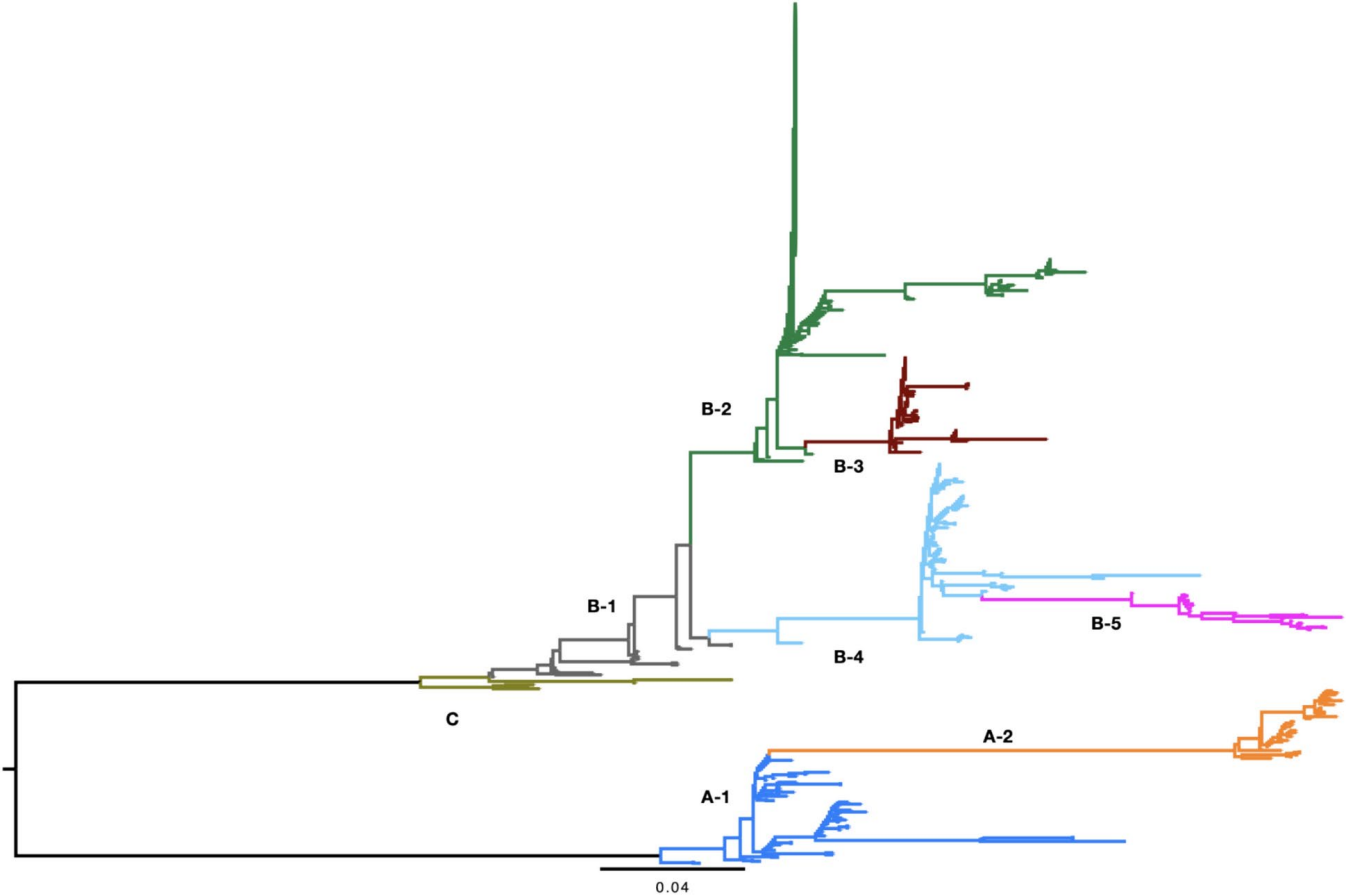
Midpoint-rooted phylogenetic tree of CynVolERV-A, CynVolERV-B, and CynVolERV-C long terminal repeat (LTR) sequences. A maximum-likelihood phylogenetic tree was constructed using the GTR gamma substitution model. CynVolERV-A LTR subgroups are labeled A1 and A2, CynVolERV-B LTR subgroups are labelled B-1 to B-5, and CynVolERV-C LTRs are labelled C.

The proviral 5’ and 3’ LTRs are identical in sequence when the provirus is formed in the genome, subsequently the LTRs accumulate mutations through time independently according to the mutation rate of the host. We observed limited sequence variation between proviral 5’ and 3’ LTR sequences for all proviruses in all three proviral groups. Within proviral groups we observed a more complex LTR sequence evolution, specifically proviral 5’ and 3’ LTRs, when compared to those of other proviruses, displayed considerable sequence variation, especially indels of different lengths and different combinations of such indels (Fig. S3). Phylogenetic analysis of all proviral LTR sequences revealed two distinct LTR clades for CynVolERV-A proviruses, five distinct clades for CynVolERV-B proviruses and one clade for CynVolERV-C proviruses (Fig. 2). Phylogenetic separation of each provirus’ LTR sequences into different clades was due to different indel combinations and different indel sizes present even among closely related proviral sequences. While different phylogenetic clades were formed due to variability of sequences among LTRs of the same provirus it appears that there were very few nucleotide differences among sequences forming a clade. At the same time a few proviruses harbor unique indels within their 5’ and 3’LTRs (Table S3). While there are clearly primarily indel-based LTR subgroups it was sometimes difficult to assign LTRs to one or another subgroup because of additional indels within the LTRs.

### Screening of sequences from Cynocephalus volans museum and modern samples reveals CynVolERV highly similar sequences in only some samples

**Fig. 3:**
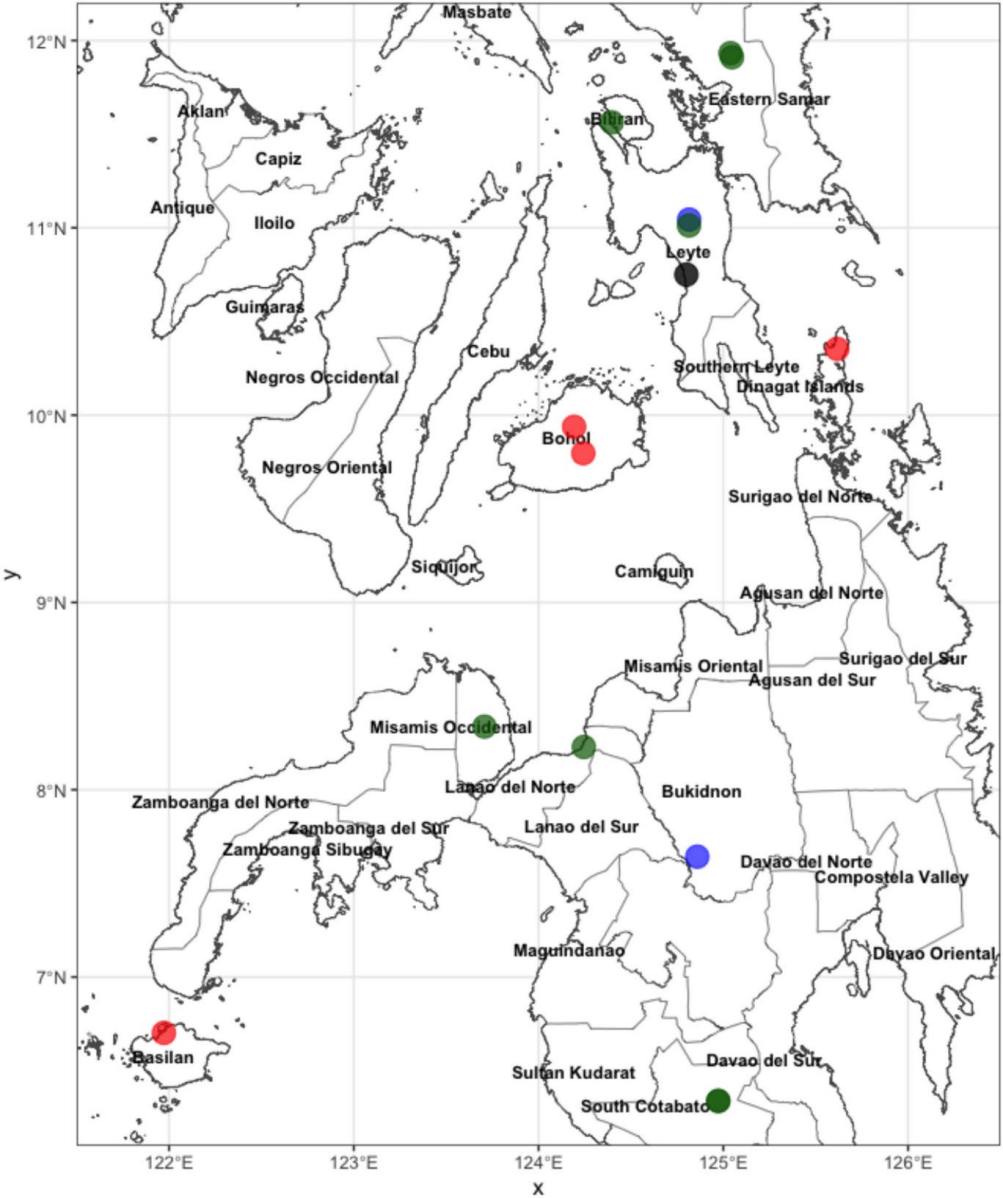
Philippines map illustrating collection points of modern and museum samples screened in this study. A black dot illustrates the sample used for sequencing and assembling the *Cynocephalus volans* genome. The same sample was also used to generate transcriptome data from heart tissue. Blue dots represent samples that had high coverage of all four consensus sequences of CynVolERVs, that is, sequence data from those samples could generate complete consensus sequences of all four ERVs. Green dots represent *Cynocephalus volans* samples that had lower coverage of mapped reads to CynVolERV proviral consensus. Red dots indicate samples without reads matching CynVolERV sequences.

To verify the presence of the identified CynVolERV proviruses in more than the one *Cynocephalus volans* sample that was used to sequence and assemble the *C. volans* genome, sequencing data from 13 *C. volans* museum samples and 1 *C. volans* modern sample were obtained from the NCBI Sequence Read Archive (SRA) (Table 1). The samples were collected from a variety of locations in the Philippines at different time points (Table 1, Fig. 3). Obtained SRA data were either from a low-coverage whole genome sequencing approach, or were sequenced after a hybrid capture enrichment procedure. Obtained fastq data were mapped to CynVolERV A-C reference sequences using Bowtie2^30^. Out of 14 SRA datasets that were screened for CynVolERVs, samples PDH_3306 and AMNH_187861 produced high quality consensus sequences for all four proviral sequences, eight samples had ~ 150 to 350 positive hits for some CynVolERV proviral genes, and four samples were negative (NMNH_462160, NMNH_462160, AMNH_85042, AMNH_16219) (Table 1, Fig. 3).

**Table 1:** *Cynocephalus volans* museum and modern sample-derived data obtained from NCBI sequence read archive (SRA).

SRA ID	Sample_ID	SRA_accession	Date_of_collection	Geographic_Location	Sample type	Sequencing	CynVolERVs presence
mCynVol1	mCynVol1	SRX23426437	1987	Layten Island	Modern	WGS	TRUE
CV0_02	PDH_3306	SRX1974305	N/A	Mindanao	Modern	WGS	TRUE
CVO_03	AMNH_24958	SRX1854847	1905	Samar Island	Museum	WGS	TRUE
CVO_06	AMNH_24981	SRX1854858	1905	Samar Island	Museum	WGS	TRUE
CVO_07	AMNH_85042	SRX1854869	N/A	Bohoi	Museum	WGS	FALSE
CVO_08	AMNH_203257	SRX1854909	1962	Tupi, Mindanao	Museum	Targeted NGS	TRUE
CVO_09	AMNH_16219	SRX1854891	N/A	Bohoi	Museum	WGS	FALSE
CVO_10	AMNH_203258	SRX1854902	1962	Tupi, Mindanao	Museum	WGS	TRUE
CVO_13	AMNH_187860	SRX1854912	1961	Layten Island	Museum	WGS	TRUE
CVO_15	AMNH_187861	SRX1854911	1961	Layten Island	Museum	Targeted NGS	TRUE
CVO_17	NMNH_219289	SRX1854914	1918	Misamis Occidental, Mindanao	Museum	WGS	TRUE
CVO_19	NMNH_578084	SRX1854848	1987	Samar Island	Museum	WGS	TRUE
CVO_21	NMNH_144663	SRX1854849	1906	Isabella City, Basilan (Island)	Museum	Targeted NGS	FALSE
CVO_22	NMNH_113493	SRX1854850	1901–1909	Iligan city ,Mindanao	Museum	WGS	TRUE
CVO_24	NMNH_462160	SRX1854851	1975	Loreto, Dinagat Island	Museum	Targeted NGS	FALSE

### Phylogenetic analysis of proviral genomes reveals close relationship to GALV-like sequences

**Fig. 4:**
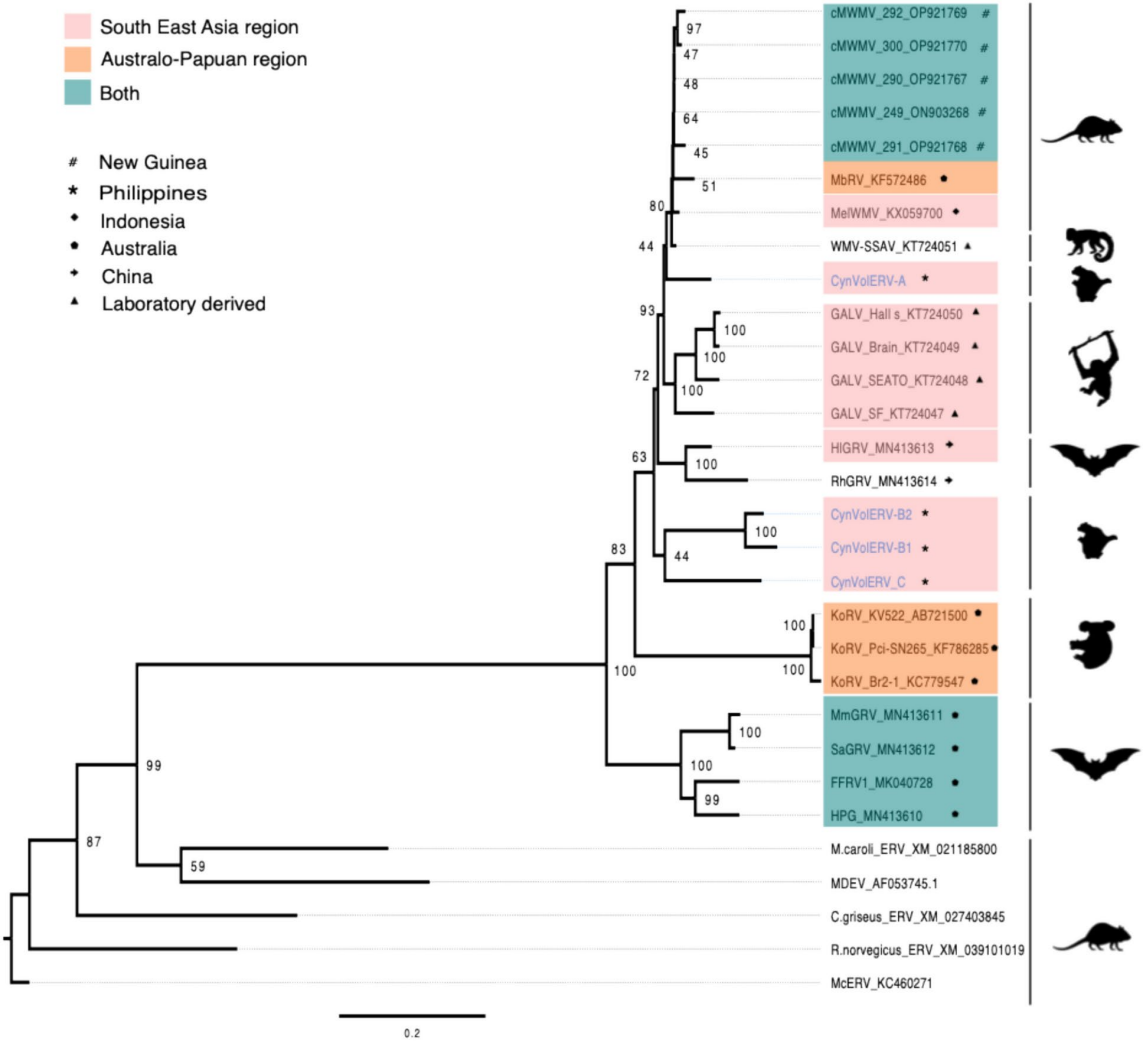
Phylogenetic placement of CynVolERV-A, CynVolERV-B1, CynVolERV-B2, and CynVolERV-C consensus nucleotide sequences within Retroviridae. Biogeographic distribution of species in the CynVolERV clade is also illustrated with species of the South East Asia regions being highlighted in pink, species that are distributed in Australo-Papua region being highlighted in orange, and species that can be found in both geographic regions being highlighted in green. Specific sampling information for the CynVolERV clade sequences is also illustrated. A RAxML phylogenetic tree was constructed using the GTR gamma substitution model with 20 maximum likelihood searches and 200 rapid bootstrap replicates. McERV sequence was used as an outgroup.

We generated multiple alignments, using MAFFT^24^, of sequence collections compiled in-house and from BLAST searches using CynVolERV-A and CynVolERV-B proviral consensus sequences and protein sequences of retroviral genes, as predicted by RetroTector^23^. Maximum-likelihood phylogenetic analysis was performed using *Mus caroli* endogenous virus (McERV) as an outgroup for both nucleotide and protein alignments. Nucleotide phylogenetic analysis placed CynVolERV-A, CynVolERV-B, and CynVolERV-C in the GALV-like clade along with GALV, WMV and cMWMV. CynVolERV-A was basal to the WMV-SSAV-related sequences whereas CynVolERV-B and CynVolERV-C formed a sister group to the entire GALV-like clade (Fig. 4). Phylogenetic trees based on RetroTector-predicted proteins did not appear to resolve as well as the nucleotide-based whole genome phylogeny (Fig. S4). A *gag*-derived tree was polytomous among the GALV-like sequences most likely due to lack of informative sites. As for the Gag protein phylogeny, CynVolERV-B protein sequences were basal to the GALV-like sequences, while CynVolERV-A sequences clustered in the same clade as the cMWMV sequences in a branch within the polytomous GALV-like sequences, a placement that is consistent with the nucleotide phylogeny. The CynVolERV-C Gag protein did not cluster with CynVolERV-B but was placed inside of the FFRV1 branch within the GALV-like polytomy (Fig. S4). Pol-based phylogenetic analysis likewise produced a polytomy with the GALV-like sequences, and placed CynVolERV-A, CynVolERV-B, and CynVolERV-C within the GALV-like clade (Fig. S4). Finally, CynVolERV-A Env was a sister group of the GALV clade of sequences, while CynVolERV-B2 and CynVolERV-C Env were placed within the polytomy that included KoRV sequences and GALV-like sequences (Fig. S4). Based on nucleotide-based and protein-based phylogenies we can conclude with strong confidence that the identified proviruses are related to the GALV-like sequence clade.

### Cynocephalus volans proviruses are evolutionarily young

**Fig. 5:**
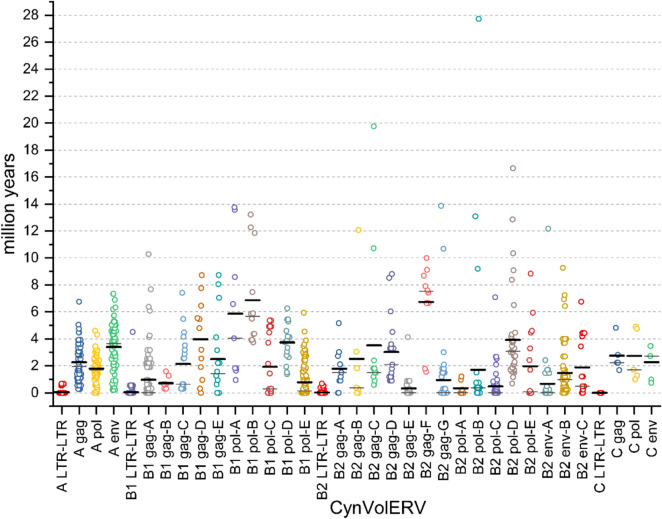
Evolutionary age estimates of individual CynVolERV proviruses. Evolutionary ages were estimated based on proviral 5’/3'LTR nucleotide divergence and nucleotide divergence of proviral *gag*, *pol* and *env* genes from subgroup-specific consensuses. Subgroups were defined for more accurate age estimates. Estimated ages of individual proviruses are indicated by circles, average and median ages are indicated by bold and thin, respectively, horizontal lines.

We used two different methods for estimating evolutionary ages of CynVolERV proviruses. First, ages were estimated based on nucleotide divergences between *gag*, *pro*, *pol* and *env* genes of CynVolERV subgroups compared to subgroup-specific majority-rule consensus sequences, based on manually curated multiple sequence alignments generated by MAFFT^24^. Due to the lack of a specific *Cynocephalus volans* mutation rate we used the mammalian mutation rate of 2.2 × 10^−9^ per base pair per year for age estimates^31^. Sequences that did not properly align were considered as outliers and excluded from age estimation analysis. Mean calculated ages per proviral gene are summarized in Table 2, individual provirus ages are depicted in Fig. 5.

**Table 2:** Estimates of evolutionary ages of CynVolERV provirus subgroups^*^.

	Proviral LTR age estimation	*Gag*	*Pol*	*Env*
**CynVolERV-A**	~ 0.10 (± 0.34)	~ 2.27 (± 1.38)	~ 1.81 (± 0.90)	~ 3.40 (± 1.73)
**CynVolERV-B1**	~ 0.09 (± 0.70)	~ 0.72 (± 0.48)	~ 5.87 (± 5.09)	
		~ 0.97 (± 1.88)	~ 6.87 (± 9.27)	
		~ 2.14 (± 2.13)	~ 1.93 (± 2.26)	
		~ 3.97 (± 1.96)	~ 3.73 (± 1.46)	
		~ 2.50 (± 2.98)	~ 0.78 (± 1.19)	
**CynVolERV-B2**	~ 0.05 (± 0.22)	~ 1.79 (± 1.50)	~ 0.35 (± 0.52)	~ 0.67 (± 1.93)
		~ 2.52 (± 4.37)	~ 0.55 (± 1.61)	~ 1.48 (± 1.80)
		~ 2.17 (± 2.74)	~ 0.48 (± 1.11)	~ 1.88 (± 2.21)
		~ 3.03 (± 2.44)	~ 3.93 (± 3.37)	
		~ 0.33 (± 0.80)	~ 1.96 (± 2.74)	
		~ 6.74 (± 2.87)		
		~ 0.95 (± 2.41)		
**CynVolERV-C**	~ 0.00 (± 0.00)	~ 2.76 (± 1.22)	~ 2.74 (± 1.94)	~ 2.28 (± 1.33)

*For the sake of more accurate age estimates, CynVolERV-B1 *gag* and *pol* genes were split into five subgroups, CynVolERV-B2 *gag* into seven subgroups, *pol* into five subgroups and *env* into three subgroups. Ages and standard deviations are given in millions of years.

Second, proviral ages were estimated using sequence divergence between proviral 5’ and 3’ LTR sequences. As mentioned above, proviral 5’ and 3’ LTRs are identical in sequence when a provirus is formed initially. The LTRs will subsequently accumulate mutations independently over time at the host mutation rate. The number of nucleotide differences between the 5’ and 3’ LTR in combination with the host mutation rate can be used to estimate proviral ages. Using again the average mammalian mutation rate^31^ and proviral 5’–3' LTR distances we calculated the evolutionary age of each provirus (Tables S4-S7). CynVolERV proviral mean evolutionary ages are presented in Table 2, individual proviral age distribution for both LTR-LTR and proviral genes are illustrated in Fig. 5.

Age estimates from both methods illustrated that the identified ERVs are evolutionarily very young. Gene-based age estimates ranged from approx. 0.35 myr old to approx. 6.87 myr old, while the proviral LTR age estimates ranged from approx. 0.00 myr to approx. 0.10 myr old (Table 2). Proviral gene-based age estimation had a higher age range due to a higher sequence variability among the examined sequences (Fig. 5). LTR to LTR age estimates were less variable due to the lack of mutations between the LTRs of the same provirus (Fig. 5). Taking into account estimated ages of CynVolERV proviruses greater than zero myr (Tables S4-S18) ages of those proviruses were estimated as low as around 0.14 myr. It therefore appears reasonable to speculate that the youngest CynVolERV proviruses are a few hundreds of thousands of years old, or younger.

We note that gene conversion events between proviral LTRs likely contribute to decreased sequence variation between proviral LTRs, thus the particular method for proviral age estimation can be biased towards lower evolutionary ages^32,33^. We also note that, in the light of above age estimates having established an evolutionarily relatively very recent formation of CynVolERV proviruses, we did not estimate proviral ages based on nucleotide divergence of CynVolERV LTRs to LTR consensuses. Besides overall very few nt differences between proviral LTRs, the amount of indel variations seen for CynVolERV LTRs would require, in principle, many different consensuses compared to very few or even only two proviral LTRs, that furthermore often would be (almost) identical in sequence to the particular consensus. In any case, the above estimate of the youngest CynVolERV proviruses being a few hundreds of thousands of years old, or younger, establishes that CynVolERVs resulted from a relatively very recent germ line colonization event.

## Discussion

Wallace’s line is a biogeographical barrier that intersects the Indian ocean, between the Sunda Arc Islands of Bali and Lombok, passing between Borneo and Sulawesi, and ending to the east of the Philippines. It was first described by Alfred Wallace and it represents a distributional boundary for almost all fauna with different species being found on each side of the line^34^. GALV/KoRV-like endogenous retroviruses have been identified in several rodent and bat species from the Australo-Papuan side of the Wallace’s line, while on the Southeast Asian side of the Wallace’s line closely related partial sequences were identified only in two Chinese endemic bat species. Since the isolation of GALV sequence in the SEATO medical research facility in Thailand in the 1960s and 1970s from captive gibbons, most likely due to iatrogenic transmission, no other GALV related sequence have been isolated from gibbons or other mammals in the South East Asia^15^. Screening of 31 Southeast Asia origin mammalian genome species for KoRV/GALV-like viruses identified endogenous retroviral sequences with high similarity to GALV exclusively in the genome of *Cynocephalus volans*, also called the Philippine flying lemur^35^. Screening available genomic sequences of the related Sunda flying lemur ,*Galeopterus variegatus*, failed to identify KoRV/GALV like sequences*.* The ERV sequences identified in the *Cynocephalus volans* genome were named CynVolERV-A, CynVolERV-B1, CynVolERV-B2, and CynVolERV-C. These ERVs identified in *C. volans* represent, to the best of our knowledge, the first example of a primate relative having been infected relatively recently, in an evolutionary timescale context, with a GALV-like retrovirus in the wild. Phylogenetic analysis of the identified ERVs placed the CynVolERV-B1/B2/C groups of sequences as a sister clade of all GALV-like sequences and CynVolERV-A basal to the WMV-like group of GALV sequences.

CynVolERV proviral sequence analysis demonstrated the presence of all retroviral genes in the majority of sequences. This is consistent with age estimations for the various subgroups of CynVolERV suggesting the entire group is evolutionarily young, with most proviral 5’/3' LTRs showing very few or no nucleotide differences. Notwithstanding potential gene conversion events between proviral LTRs biasing towards younger provirus ages^32,36^ our data indicate that CynVolERV proviruses either formed very recently in evolution and/or they are actively proliferating. That more unique LTR groups could be identified than overall proviral groups suggests that for some CynVolERVs, multiple unique germline colonizations took place. This is similar to KoRV which likely also is the result of multiple independent germline colonization events^37^. Transcriptome analysis of heart tissue from a Philippine flying lemur individual demonstrated transcripts from all four CynVolERV subgroups. That finding supports continuing activity of CynVolERV proviral sequences, and that CynVolERV-A, CynVolERV-B2, and CynVolERV-C could potentially produce infectious viral particles. However, transcribed CynVolERV-B1 may lack such a capacity due to a missing 133 bp *pol* region and a 1487 bp deletion within the *env* gene, though trans-complementation between CynVolERV subgroups is conceivable. Proliferation of the B1 lineage, despite its proviruses lacking most of the *env* gene region, can be explained by *env*-less 'ERV groups often becoming genomic superspreaders . This behaviour of *env*-less ERVs is widespread among vertebrates and is likely a general principle of the endogenization process^38^.

Further screening of *Cynocephalus volans* modern and museum samples from multiple time points and various locations in Philippines illustrated the presence of the CynVolERVs in multiple *Cynocephalus volans* individuals. There were, however, four samples that did not appear to have reads related to CynVolERV. Negative samples were collected from Bolol, Dinagat, and Basilan islands. Negative results could be explained partially by the sample processing method. Two of the negative samples (NMNH 144,663 and NMNH 462,160) were first enriched for a specific gene panel before being sequenced. The enrichment method may have depleted CynVolERV sequences from those samples. However, that explanation cannot apply to the other two CynVolERV-negative samples that were subjected to whole genome sequencing with high sequence output. Another explanation for the negative results in some individuals could be that *Cynocephalus volans* animals in Bolol, Dinagat, and Basilan islands were not infected by exogenous CynVolERV and the particular *Cynocephalus volans* populations therefore are CynVolERV-free. However, further sampling is needed to determine the CynVolERV status in *Cynocephalus volans* individuals with respect to geographic region.

Our results suggest evolutionarily recently or currently circulating KoRV/GALV-like retroviruses are present in Southeast Asian mammals and that the KoRV/GALV clade is not as restricted to the Australo-Papuan region as thought. Of particular note, almost all discovered KoRV and GALV-like sequence relatives, when not basal to the overall clade, are very often closely related to WMV, whereas retroviruses closely related to KoRV or the other GALV strains have not been identified to date. Whether this represents under-sampling of taxa from the region or exogenous WMV-like viruses being the only representatives of the clade still being transmitted exogenously remains unclear. As more mammalian genome sequences become available, the extent of the viral leakiness of Wallace’s Line and the overall distribution and diversity of the GALV-KoRV clade should become clearer.

## Material and methods

### Genome screening

Southeast Asian mammalian genome sequences (Table S1) were downloaded from NCBI Datasets database^21^. GALV, KoRV, and cMWMV (KT724047, KF786285, ON903268) reference sequences were used as seeds to BLAST search the corresponding genomes using both megablast and blastn algorithms with default parameters^39^. Positive BLAST hits coordinates + /- 10,000 bp were extracted from genome sequences using bedtools v2.31.0 getfasta algorithm^40^. Extracted sequences were aligned using MAFFT v7.450 with default settings^24^. Resulting alignments were manually curated, annotated with Geneious Prime v2024.0.4 using a gammaretroviral database. Majority-rule consensus sequences were generated using Geneious Prime v2024.0.4. Repeatmasker v4.1.6 and Retrotector v1.0.1 scripts^22,23^ were used to define the proviral gene regions and LTRs of the majority consensus sequences. Putative protein sequences were generated from the majority consensus sequence using Retrotector v1.0.1, while the conserved protein domains were identified using the NCBI Conserved Domain Database^23,25^.

### CynVolERV expression analysis

To investigate expression of CynVolERV proviruses, we made use of an Illumina RNA-seq paired-end sequence dataset (SRR27761458) previously generated from a heart tissue sample from a male *Cynocephalus volans* individual. We aligned approximately 134 million reads in that dataset to consensus sequences of CynVolERV-A, CynVolERV-B1, CynVolERV-B2, and CynVolERV-C using RNA STAR^28^ at usegalaxy.eu with the index^41^ built without a gene model and otherwise standard parameters. Reads uniquely mapping to CynVolERV consensus sequences were extracted from RNA STAR log files.

### Phylogenetic analysis

Multiple alignments of gammaretroviral nucleotide and amino acid sequences were generated using MAFFT v 7.450^42^ followed by manual curation. For the phylogenetic analysis the majority-rule consensus proviral sequences, Retrotector-predicted proviral protein sequences of major retroviral genes (*gag*, *pol*, and *env*) of CynVolERV subgroups, and the following related sequences obtained from GenBank were used: *Mus caroli* ERV (XM_021185800), *Cricetulus griseus* ERV (XM_027403845), McERV (KC460271), MDEV (AF053745.1), *Rattus norvegicus* ERV (XM_039101019), HPG (MN413610), FFRV1 (MK0400728), MmGRV (MN413611), SaGRV (MN413612), RhGRV (MN413614), KoRV_Pci-SN265 (KF786285), KoRV KV522 (AB721500), KoRV Br2-1 (KC779547), HIGRV (MN413613), MelWMV (KX059700), WMV-SSAV (KT724051), GALV-SF (KT724047), GALV-SEATO(KT724048), GALV-Brain (KT724049), GALV-Hall’s (KT724050), cMWMV_249 (ON903268), cMWMV_290 (OP921767), cMWMV_291 (OP921768), cMWMV_292 (OP921769), cMWMV_300 (OP921770), and MbRV (KF572486). Phylogenetic analysis was performed using RAxML v8 maximum-likelihood inference program with 20 maximum likelihood searches and 200 rapid bootstrap replicates for both nucleotide and amino acid alignments. General time distribution (GTR) model with gamma distribution and invariable sites was used for the nucleotide alignment. FLU model with gamma distribution was used for protein alignments^43^.

### CynVolERV provirus age estimation

CynVolERV proviral ages were estimated using two different approaches. First, if more accurate, proviral gene sequences were grouped into subgroups based on sequence similarity. Majority-rule consensus sequences were generated from resulting alignments for each group or subgroup. Kimura-2-parameter (K2P) corrected distances^44^ were calculated per subgroup and gene, with CpG dinucleotide positions excluded from the analysis due to their higher mutation rate because of spontaneous deamination of 5-methylcytosine^45^. Resulting alignments were manually curated and nucleotide divergences from the subgroup-specific majority rule consensus sequence were calculated. Ages were calculated using sequence divergences from their consensus assuming a molecular clock (Table S8-S18). Second, sequence divergences between proviral 5’ and 3’LTRs were determined, likewise excluding CpG sites. Proviral ages were calculated using $T=D/(2 x M)$, where D is the K2P-corrected distance between the 5’ and 3’ proviral LTR^46^ (Table S4-S7). Mean and standard deviation were calculated for both age estimation approaches. A previously reported mammalian mutation rate of 0.0022/nt/myr^31^ was used.

## Supplementary Information

Supplementary Information 1.

Supplementary Information 2.

Supplementary Information 3.

Supplementary Information 4.

Supplementary Information 5.

Supplementary Information 6.

## Data Availability

The reference *Cynocephalus volans* genome is available at GenBank datasets: GCA_027409185.1 [https://www.ncbi.nlm.nih.gov/datasets/genome/GCF_027409185.1/].
