# Supplementary material for: Crossing Wallace’s line: an evolutionarily young gibbon ape leukemia virus like endogenous retrovirus identified from the Philippine flying lemur (Cynocephalus volans)
Source: Sci Rep. 2025 Mar 21;15:9790. doi: 10.1038/s41598-025-94582-1 (PMC11928578; doi:10.1038/s41598-025-94582-1)
Supplement: Supplementary file 2 — Supplementary Information 2. [file 41598_2025_94582_MOESM2_ESM.pdf]

Crossing Wallace's Line: A Gibbon Ape Leukemia Virus like retrovirus identified from the Philippine flying lemur (*Cynocephalus Volans*)
Kyriakos Tsangaras[1,4], Jens Mayer [2], Alex D. Greenwood[3]x*

Affiliations:
[1] University of Nicosia, Department of Life and Health Sciences, Nicosia, Cyprus
[2] Institute of Human Genetics, Medical Faculty, University of Saarland, Homburg, Germany
[3] Department of Wildlife Diseases, Leibniz Institute for Zoo and Wildlife Research (IZW), Berlin, Germany
[4] Department of Cardiovascular Genetics and the Laboratory of Forensic Genetics , Cyprus Institute of Neurology and Genetics, Nicosia, Cyprus
[x] School of Veterinary Medicine, Freie Universität Berlin, Berlin, Germany

* Corresponding author: greenwood@izw-berlin.de

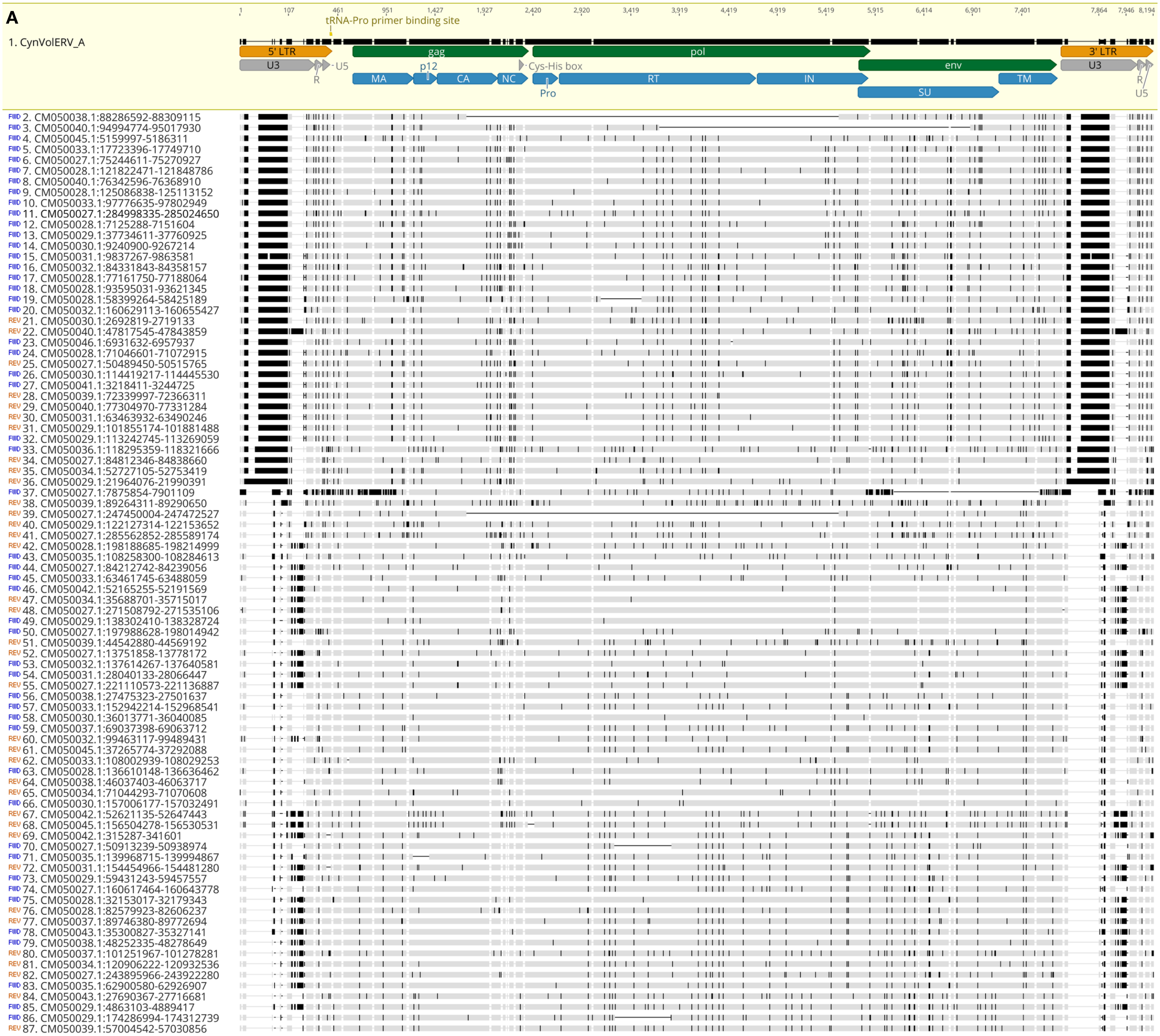

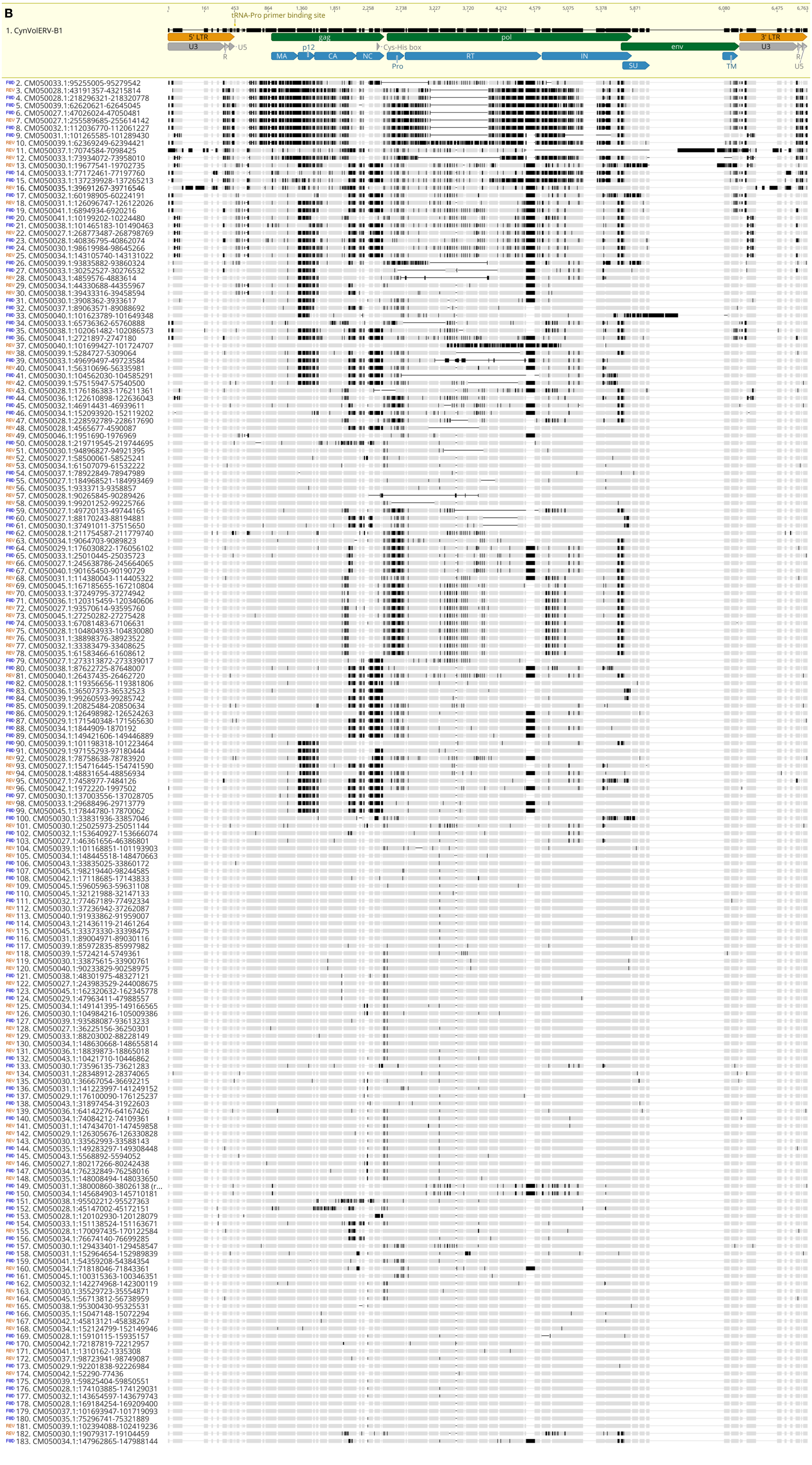
B
1 161 453 864 1,360 1,851 2,258 2,738 3,227 3,720 4,212 4,579 5,075 5,378 5,871 6,080 6,475 6,763
tRNA-Pro primer binding site
1. CynVolERV-B1
5' LTR
gag
pol
env
3' LTR
U3
R
U5
p12
MA
CA
NC
Cys-His box
Pro
RT
IN
SU
TM
FWD 2. CM050033.1:95255005-95279542
REV 3. CM050028.1:43191357-43215814
FWD 4. CM050028.1:218296321-218320778
FWD 5. CM050039.1:62620621-62645045
FWD 6. CM050027.1:47026024-47050481
REV 7. CM050027.1:255589685-255614142
FWD 8. CM050032.1:112036770-112061227
FWD 9. CM050031.1:101265585-101289430
REV 10. CM050039.1:62369249-62394421
REV 11. CM050037.1:7074584-7098425
REV 12. CM050033.1:73934072-73958010
REV 13. CM050030.1:19677541-19702735
FWD 14. CM050033.1:77172461-77197760
FWD 15. CM050033.1:137239928-137265213
REV 16. CM050035.1:39691267-39716546
FWD 17. CM050032.1:60198905-60224191
REV 18. CM050031.1:126096747-126122026
FWD 19. CM050041.1:6894934-6920216
FWD 20. CM050041.1:10199202-10224480
FWD 21. CM050038.1:101465183-101490463
REV 22. CM050027.1:268773487-268798769
FWD 23. CM050028.1:40836795-40862074
FWD 24. CM050030.1:98619984-98645266
REV 25. CM050034.1:143105740-143131022
FWD 26. CM050039.1:93835882-93860324
FWD 27. CM050033.1:30252527-30276532
REV 28. CM050043.1:4859576-4883614
REV 29. CM050034.1:44330688-44355967
REV 30. CM050038.1:39433316-39458594
FWD 31. CM050030.1:3908362-3933617
REV 32. CM050037.1:89063571-89088692
FWD 33. CM050040.1:101623789-101649348
FWD 34. CM050033.1:65736362-65760888
FWD 35. CM050038.1:102061482-102086573
FWD 36. CM050041.1:2721897-2747180
REV 37. CM050040.1:101699427-101724707
REV 38. CM050039.1:5284727-5309064
FWD 39. CM050033.1:49699497-49723584
REV 40. CM050041.1:56310696-56335981
FWD 41. CM050030.1:104562030-104585291
REV 42. CM050039.1:57515947-57540500
REV 43. CM050028.1:176186383-176211361
FWD 44. CM050036.1:122610898-122636043
FWD 45. CM050032.1:46914431-46939611
FWD 46. CM050034.1:152093920-152119202
REV 47. CM050028.1:228592789-228617690
REV 48. CM050028.1:4565677-4590087
REV 49. CM050046.1:1951690-1976969
FWD 50. CM050028.1:219719545-219744695
REV 51. CM050030.1:94896827-94921395
REV 52. CM050027.1:58500061-58525241
REV 53. CM050034.1:61507079-61532222
FWD 54. CM050037.1:78922849-78947989
FWD 55. CM050027.1:184968521-184993469
REV 56. CM050035.1:9333713-9358857
REV 57. CM050028.1:90265845-90289426
REV 58. CM050039.1:99201252-99225766
FWD 59. CM050027.1:49720133-49744165
FWD 60. CM050027.1:88170243-88194881
FWD 61. CM050030.1:37491011-37515650
FWD 62. CM050028.1:211754587-211779740
REV 63. CM050034.1:9064703-9089823
FWD 64. CM050029.1:176030822-176056102
FWD 65. CM050033.1:25010445-25035723
REV 66. CM050027.1:245638786-245664065
FWD 67. CM050040.1:90165450-90190729
REV 68. CM050031.1:114380043-114405322
REV 69. CM050045.1:167185655-167210804
REV 70. CM050033.1:37249795-37274942
FWD 71. CM050036.1:120315459-120340606
REV 72. CM050027.1:93570614-93595760
REV 73. CM050045.1:27250282-27275428
FWD 74. CM050033.1:67081483-67106631
REV 75. CM050028.1:104804933-104830080
REV 76. CM050031.1:38898376-38923522
REV 77. CM050032.1:33383479-33408625
REV 78. CM050035.1:61583466-61608612
FWD 79. CM050027.1:273313872-273339017
FWD 80. CM050038.1:87622725-87648007
REV 81. CM050040.1:26437435-26462720
FWD 82. CM050028.1:119356656-119381806
FWD 83. CM050036.1:36507373-36532523
FWD 84. CM050039.1:99260593-99285742
FWD 85. CM050039.1:20825484-20850634
FWD 86. CM050029.1:126498982-126524263
FWD 87. CM050029.1:171540348-171565630
FWD 88. CM050034.1:1844909-1870192
FWD 89. CM050034.1:149421606-149446889
FWD 90. CM050039.1:101198318-101223464
FWD 91. CM050029.1:97155293-97180444
REV 92. CM050028.1:78758638-78783920
REV 93. CM050027.1:154716445-154741590
REV 94. CM050028.1:48831654-48856934
REV 95. CM050027.1:7458977-7484126
REV 96. CM050042.1:1972220-1997502
FWD 97. CM050030.1:137003556-137028705
REV 98. CM050033.1:29688496-29713779
FWD 99. CM050045.1:17844780-17870062
FWD 100. CM050030.1:33831936-33857046
REV 101. CM050030.1:25025973-25051144
FWD 102. CM050032.1:153640927-153666074
FWD 103. CM050027.1:46361656-46386801
REV 104. CM050039.1:101168851-101193903
REV 105. CM050034.1:148445518-148470663
FWD 106. CM050043.1:33835025-33860172
FWD 107. CM050045.1:98219440-98244585
FWD 108. CM050042.1:17118685-17143833
REV 109. CM050045.1:59605963-59631108
FWD 110. CM050045.1:32121988-32147133
FWD 111. CM050032.1:77467189-77492334
REV 112. CM050030.1:37236942-37262087
REV 113. CM050040.1:91933862-91959007
FWD 114. CM050043.1:21436119-21461264
REV 115. CM050045.1:33373330-33398475
FWD 116. CM050031.1:89004971-89030116
FWD 117. CM050039.1:85972835-85997982
REV 118. CM050039.1:5724214-5749361
REV 119. CM050030.1:33875615-33900761
REV 120. CM050040.1:90233829-90258975
FWD 121. CM050038.1:48301975-48327121
REV 122. CM050027.1:243983529-244008675
FWD 123. CM050045.1:162320632-162345778
FWD 124. CM050029.1:47963411-47988557
REV 125. CM050034.1:149141395-149166565
REV 126. CM050030.1:104984216-105009386
FWD 127. CM050039.1:93588087-93613233
REV 128. CM050027.1:36225156-36250301
REV 129. CM050033.1:88203002-88228149
REV 130. CM050034.1:148630668-148655814
REV 131. CM050036.1:18839873-18865018
FWD 132. CM050043.1:10421710-10446862
FWD 133. CM050030.1:73596135-73621283
REV 134. CM050031.1:28348912-28374065
REV 135. CM050030.1:36667054-36692215
FWD 136. CM050031.1:141223997-141249152
FWD 137. CM050029.1:176100090-176125237
FWD 138. CM050043.1:31897454-31922603
REV 139. CM050036.1:64142276-64167426
FWD 140. CM050034.1:74084212-74109361
REV 141. CM050031.1:147434701-147459858
REV 142. CM050029.1:126305676-126330828
REV 143. CM050030.1:33562993-33588143
FWD 144. CM050035.1:149283297-149308448
FWD 145. CM050043.1:5568892-5594052
REV 146. CM050027.1:80217266-80242438
FWD 147. CM050034.1:76232849-76258016
REV 148. CM050035.1:148008494-148033650
FWD 149. CM050031.1:38000860-38026138 (r...
FWD 150. CM050034.1:145684903-145710181
FWD 151. CM050038.1:95502212-95527363
FWD 152. CM050028.1:45147002-45172151
FWD 153. CM050028.1:120102930-120128079
FWD 154. CM050033.1:151138524-151163671
REV 155. CM050028.1:170097435-170122584
FWD 156. CM050034.1:76674140-76699285
FWD 157. CM050030.1:129433401-129458547
FWD 158. CM050031.1:152964654-152989839
FWD 159. CM050041.1:54359208-54384354
REV 160. CM050034.1:71818046-71843361
FWD 161. CM050045.1:100315363-100346351
FWD 162. CM050032.1:142274968-142300119
REV 163. CM050030.1:35529723-35554871
REV 164. CM050045.1:56713812-56738959
REV 165. CM050038.1:95300430-95325531
FWD 166. CM050035.1:15047148-15072294
REV 167. CM050042.1:45813121-45838267
REV 168. CM050034.1:152124799-152149946
FWD 169. CM050028.1:15910115-15935157
FWD 170. CM050042.1:72187819-72212957
REV 171. CM050041.1:1310162-1335308
REV 172. CM050037.1:98723941-98749087
FWD 173. CM050029.1:92201838-92226984
REV 174. CM050042.1:52290-77436
FWD 175. CM050039.1:59825404-59850551
FWD 176. CM050028.1:174103885-174129031
FWD 177. CM050032.1:143654597-143679743
FWD 178. CM050028.1:169184254-169209400
FWD 179. CM050037.1:101693947-101719093
FWD 180. CM050035.1:75296741-75321889
REV 181. CM050039.1:102394088-102419236
REV 182. CM050030.1:19079317-19104459
FWD 183. CM050034.1:147962865-147988144

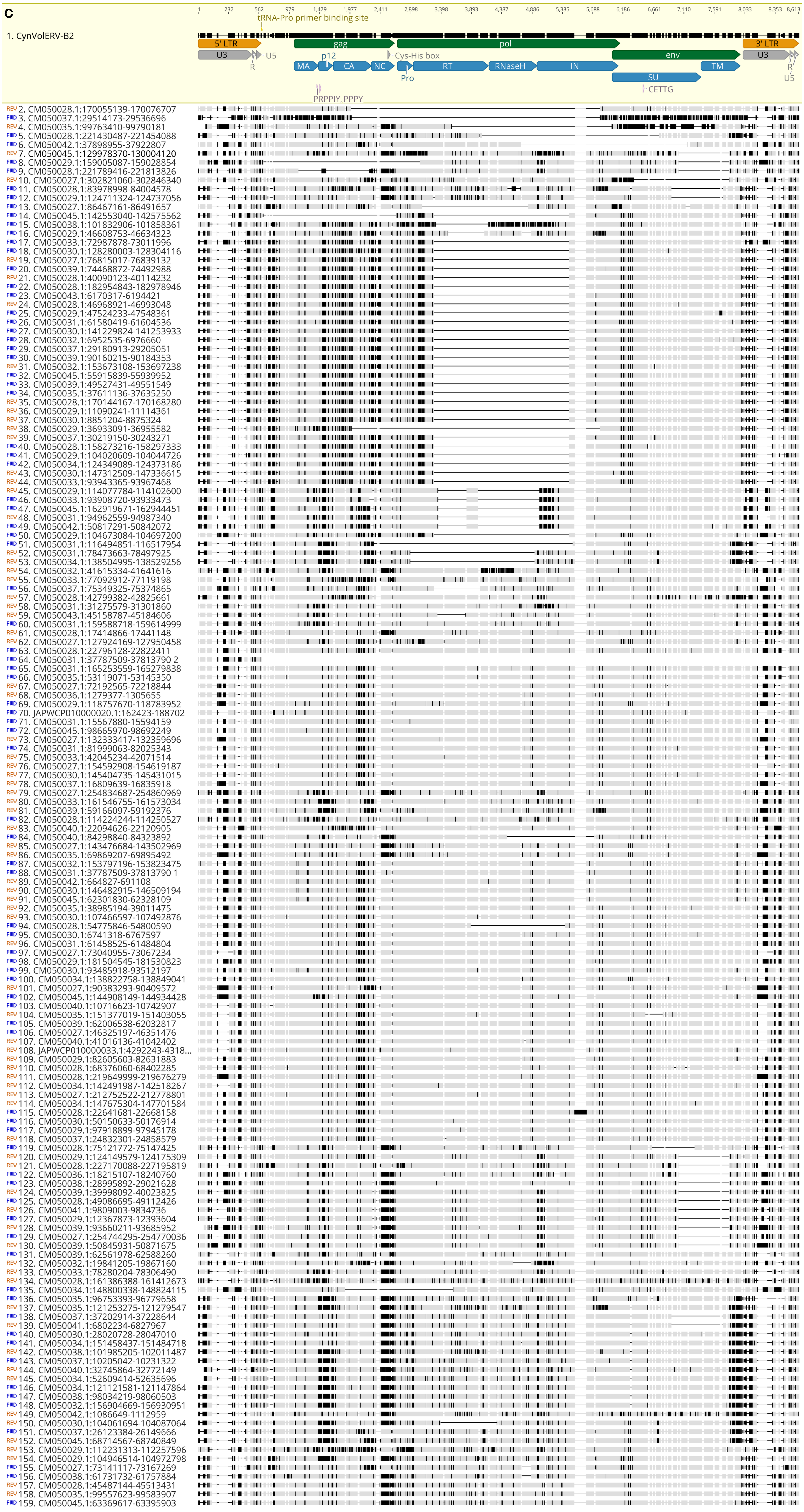
C
1 232 562 979 1,479 1,977 2,411 2,898 3,398 3,893 4,387 4,886 5,385 5,688 6,186 6,661 7,110 7,591 8,033 8,353 8,613
tRNA-Pro primer binding site
1. CynVolERV-B2
5' LTR
gag
pol
3' LTR
U3
U5
R
p12
Cys-His box
env
U3
MA
CA
NC
RT
RNaseH
IN
TM
Pro
SU
R
U5
CETTG
PRPPIY, PPPY
REV 2. CM050028.1:170055139-170076707
FWD 3. CM050037.1:29514173-29536696
REV 4. CM050035.1:99763410-99790181
FWD 5. CM050028.1:221430487-221454088
FWD 6. CM050042.1:37898955-37922807
REV 7. CM050045.1:129978370-130004120
FWD 8. CM050029.1:159005087-159028854
FWD 9. CM050028.1:221789416-221813826
REV 10. CM050027.1:302821060-302846340
FWD 11. CM050028.1:83978998-84004578
FWD 12. CM050029.1:124711324-124737056
FWD 13. CM050027.1:86467161-86491657
FWD 14. CM050045.1:142553040-142575562
FWD 15. CM050038.1:101832906-101858361
FWD 16. CM050029.1:46608753-46634323
FWD 17. CM050033.1:72987878-73011996
FWD 18. CM050030.1:128280003-128304116
REV 19. CM050027.1:76815017-76839132
FWD 20. CM050039.1:74468872-74492988
REV 21. CM050028.1:40090123-40114232
FWD 22. CM050028.1:182954843-182978946
FWD 23. CM050043.1:6170317-6194421
REV 24. CM050028.1:46968921-46993048
FWD 25. CM050029.1:47524233-47548361
FWD 26. CM050031.1:61580419-61604536
FWD 27. CM050030.1:141229824-141253933
FWD 28. CM050032.1:6952535-6976660
FWD 29. CM050037.1:29180913-29205051
FWD 30. CM050039.1:90160215-90184353
REV 31. CM050032.1:153673108-153697238
FWD 32. CM050045.1:55915839-55939952
FWD 33. CM050039.1:49527431-49551549
FWD 34. CM050035.1:37611136-37635250
REV 35. CM050028.1:170144167-170168280
REV 36. CM050029.1:11090241-11114361
REV 37. CM050030.1:8851204-8875324
REV 38. CM050029.1:36933091-36955582
REV 39. CM050037.1:30219150-30243271
FWD 40. CM050028.1:158273216-158297333
FWD 41. CM050029.1:104020609-104044726
FWD 42. CM050034.1:124349089-124373186
REV 43. CM050030.1:147312509-147336615
REV 44. CM050033.1:93943365-93967468
REV 45. CM050029.1:114077784-114102600
FWD 46. CM050033.1:93908720-93933473
FWD 47. CM050045.1:162919671-162944451
REV 48. CM050031.1:94962559-94987340
FWD 49. CM050042.1:50817291-50842072
FWD 50. CM050029.1:104673084-104697200
FWD 51. CM050031.1:116494851-116517954
REV 52. CM050031.1:78473663-78497925
REV 53. CM050034.1:138504995-138529256
REV 54. CM050032.1:41615334-41641616
REV 55. CM050033.1:77092912-77119198
FWD 56. CM050037.1:75349325-75374865
REV 57. CM050028.1:42799382-42825661
REV 58. CM050031.1:31275579-31301860
REV 59. CM050043.1:45158787-45184606
FWD 60. CM050031.1:159588718-159614999
REV 61. CM050028.1:17414866-17441148
REV 62. CM050027.1:127924169-127950458
FWD 63. CM050028.1:22796128-22822411
FWD 64. CM050031.1:37787509-37813790 2
FWD 65. CM050031.1:165253559-165279838
FWD 66. CM050035.1:53119071-53145350
REV 67. CM050027.1:72192565-72218844
REV 68. CM050036.1:1279377-1305655
FWD 69. CM050029.1:118757670-118783952
FWD 70. JAPWCP010000020.1:162423-188702
FWD 71. CM050031.1:15567880-15594159
FWD 72. CM050045.1:98665970-98692249
REV 73. CM050027.1:132333417-132359696
FWD 74. CM050031.1:81999063-82025343
REV 75. CM050033.1:42045234-42071514
REV 76. CM050027.1:154592908-154619187
REV 77. CM050030.1:145404735-145431015
REV 78. CM050037.1:16809639-16835918
REV 79. CM050027.1:254834687-254860969
REV 80. CM050033.1:161546755-161573034
REV 81. CM050039.1:59166097-59192376
FWD 82. CM050028.1:114224244-114250527
REV 83. CM050040.1:22094626-22120905
FWD 84. CM050040.1:84298840-84323892
REV 85. CM050027.1:143476684-143502969
REV 86. CM050035.1:69869207-69895492
FWD 87. CM050032.1:153797196-153823475
FWD 88. CM050031.1:37787509-37813790 1
REV 89. CM050042.1:664827-691108
REV 90. CM050030.1:146482915-146509194
REV 91. CM050045.1:62301830-62328109
REV 92. CM050035.1:38985194-39011475
REV 93. CM050030.1:107466597-107492876
FWD 94. CM050028.1:54775846-54800590
FWD 95. CM050030.1:6741318-6767597
REV 96. CM050031.1:61458525-61484804
FWD 97. CM050027.1:73040955-73067234
FWD 98. CM050029.1:181504545-181530823
FWD 99. CM050030.1:93485918-93512197
FWD 100. CM050034.1:138822758-138849041
REV 101. CM050027.1:90383293-90409572
FWD 102. CM050045.1:144908149-144934428
FWD 103. CM050040.1:10716623-10742907
REV 104. CM050035.1:151377019-151403055
FWD 105. CM050039.1:62006538-62032817
FWD 106. CM050027.1:46325197-46351476
REV 107. CM050040.1:41016136-41042402
REV 108. JAPWCP010000033.1:4292243-4318...
REV 109. CM050029.1:82605603-82631883
REV 110. CM050028.1:68376060-68402285
REV 111. CM050028.1:219649999-219676279
REV 112. CM050034.1:142491987-142518267
REV 113. CM050027.1:212752522-212778801
REV 114. CM050034.1:147675304-147701584
FWD 115. CM050028.1:22641681-22668158
FWD 116. CM050030.1:50150633-50176914
FWD 117. CM050029.1:97918899-97945178
REV 118. CM050037.1:24832301-24858579
FWD 119. CM050028.1:75121772-75147425
REV 120. CM050029.1:124149579-124175309
REV 121. CM050028.1:227170088-227195819
FWD 122. CM050036.1:18215107-18240760
FWD 123. CM050038.1:28995892-29021628
REV 124. CM050039.1:39998092-40023825
FWD 125. CM050028.1:49086695-49112426
REV 126. CM050041.1:9809003-9834736
FWD 127. CM050029.1:12367873-12393604
REV 128. CM050039.1:93660211-93685952
FWD 129. CM050027.1:254744295-254770036
REV 130. CM050039.1:50845931-50871675
FWD 131. CM050039.1:62561978-62588260
REV 132. CM050032.1:19841205-19867160
REV 133. CM050033.1:78280204-78306490
REV 134. CM050028.1:161386388-161412673
FWD 135. CM050034.1:148800338-148824115
FWD 136. CM050035.1:96753393-96779658
REV 137. CM050035.1:121253275-121279547
FWD 138. CM050037.1:37202914-37228644
REV 139. CM050041.1:6802234-6827967
FWD 140. CM050030.1:28020728-28047010
FWD 141. CM050034.1:151458437-151484718
REV 142. CM050038.1:101985205-102011487
FWD 143. CM050037.1:10205042-10231322
REV 144. CM050040.1:32745864-32772149
REV 145. CM050034.1:52609414-52635696
FWD 146. CM050034.1:121121581-121147864
FWD 147. CM050038.1:98034219-98060503
FWD 148. CM050032.1:156904669-156930951
REV 149. CM050042.1:1086649-1112959
REV 150. CM050030.1:104061694-104087064
FWD 151. CM050037.1:26123384-26149666
REV 152. CM050045.1:68714567-68740849
REV 153. CM050029.1:112231313-112257596
REV 154. CM050029.1:104946514-104972798
FWD 155. CM050027.1:73141117-73167269
FWD 156. CM050038.1:61731732-61757884
REV 157. CM050028.1:45487144-45513431
REV 158. CM050035.1:99557623-99583907
FWD 159. CM050045.1:63369617-63395903

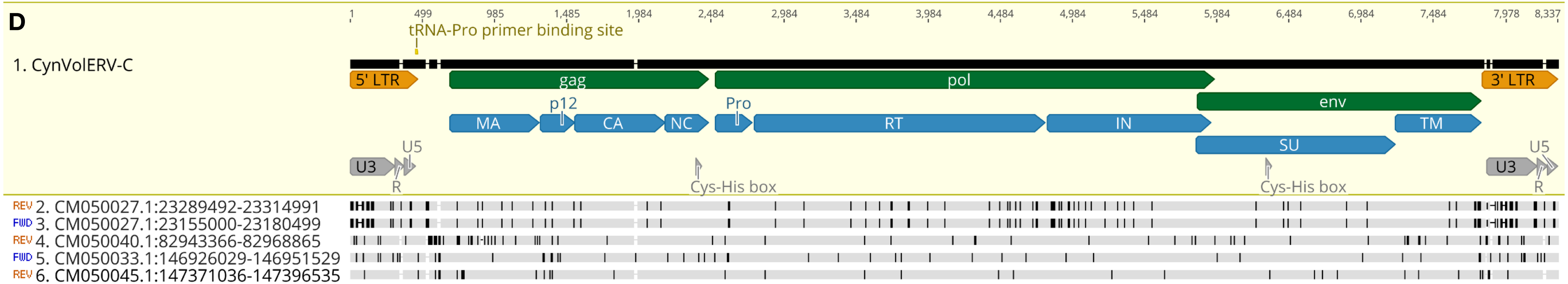

Fig S1: Multiple sequence alignments of CynVolERV-A, CynVolERV -B1, CynVolERV-B2, and CynVolERV-C proviral loci. CynVolERV sequences were identified using megablastin theCynocephalus volans reference genome (GCA_027409185.1). Proviral loci were extracted from the genome and multiply aligned using MAFFT v7. A) Proviral alignment of 86 sequences using the CynVolERV-A consensus sequence as reference. The first 36 sequences belong to the CynVolERV-A LTR2 group, while the remaining sequences belong to the CynVolERV-A LTR1 group. B) CynVolERV-B1 reference sequence aligned to 182 CynVolERV-B1proviral sequences. C) 152 CynVolERV-B2 proviral sequences aligned to CynVolERV-B2 reference sequence. D) Proviral alignment of 5 sequences against the CynVolERV-C consensus sequence. Black vertical lines indicate sequence differences compared to the reference sequence, while gray color indicates sequence identities. Note here that due to resolution, proviral regions appear more dissimilar in sequence than they actually are.

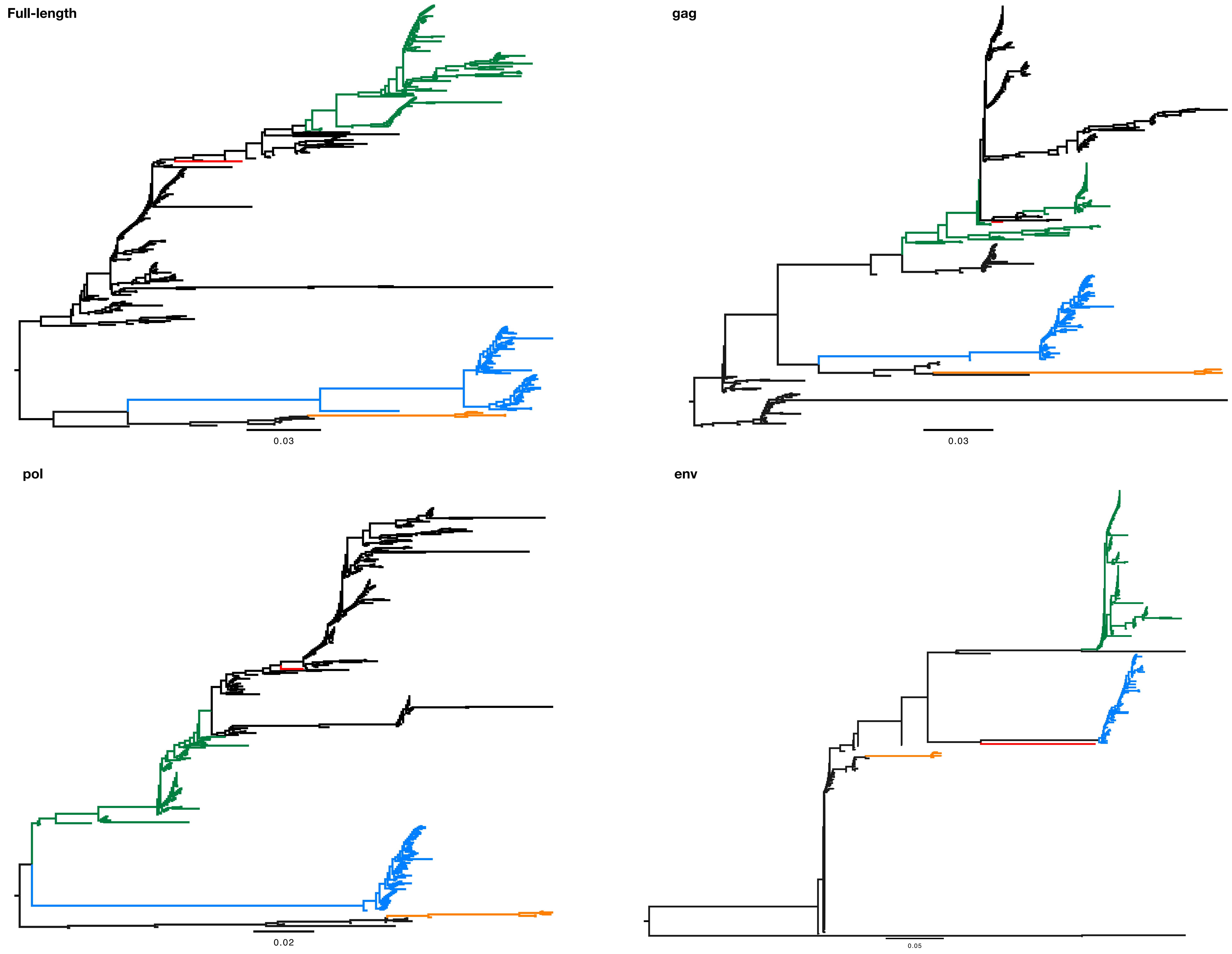

Fig S2: Phylogenetic trees for CynVolERV full-length proviral sequences and CynVolERV gag, pol end env gene regions. Trees were generated using FastTree. CynVolERV-A sequences are highlighted in blue, CynVolERV-B1 in black, CynVolERV-B2 in green, CynVolERV-C in orange. The CynVolERV-consensus sequence of all sequences included in the tree is highlighted in red each.

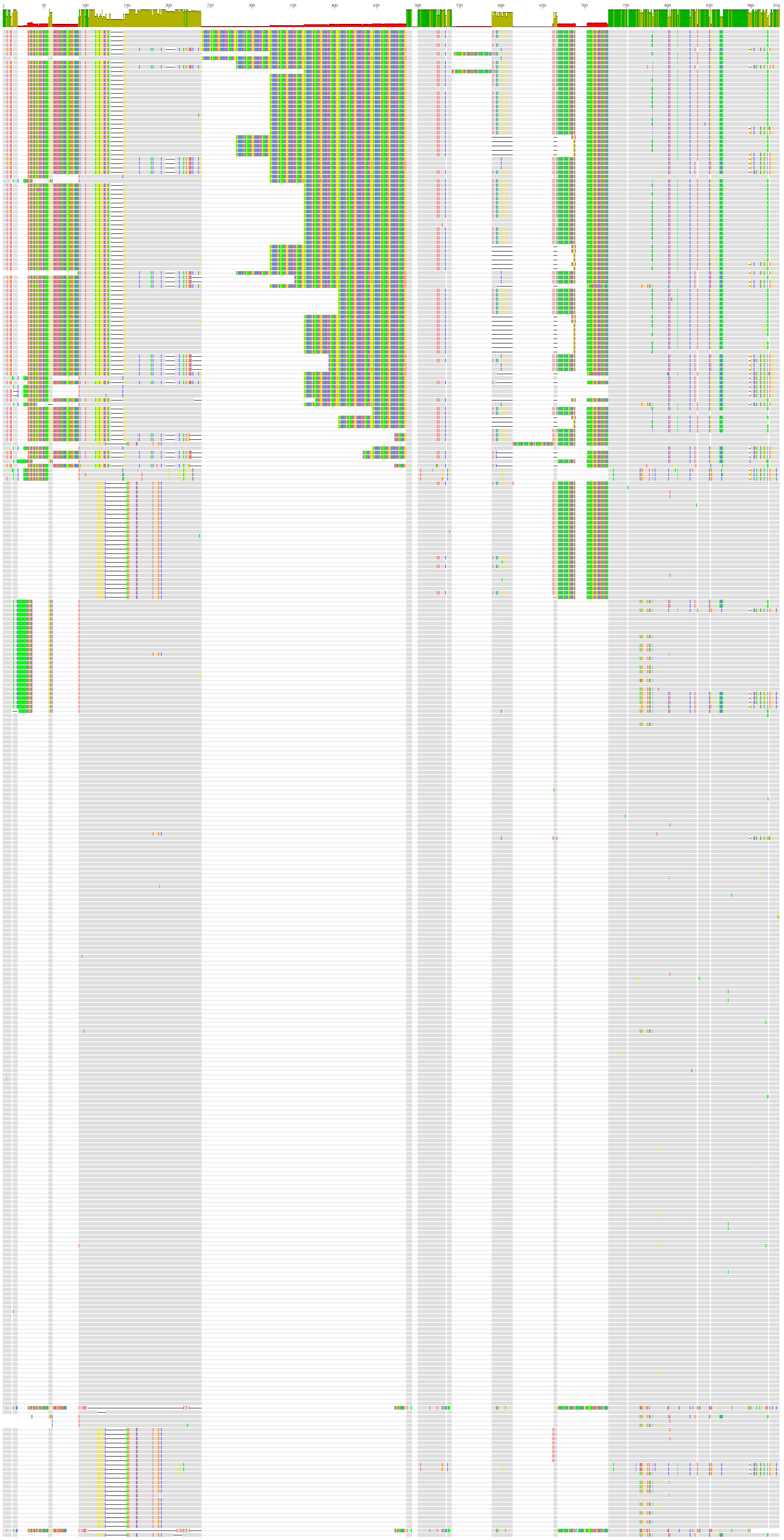

Fig S3: Multiple alignment of CynVolERV-B 5'LTRs demonstrating considerable indel variation between proviral LTRs. LTR sequences in the multiple alignment are ordered by sequence length for better depicting indel variations. Nucleotide positions identical to the consensus are in grey

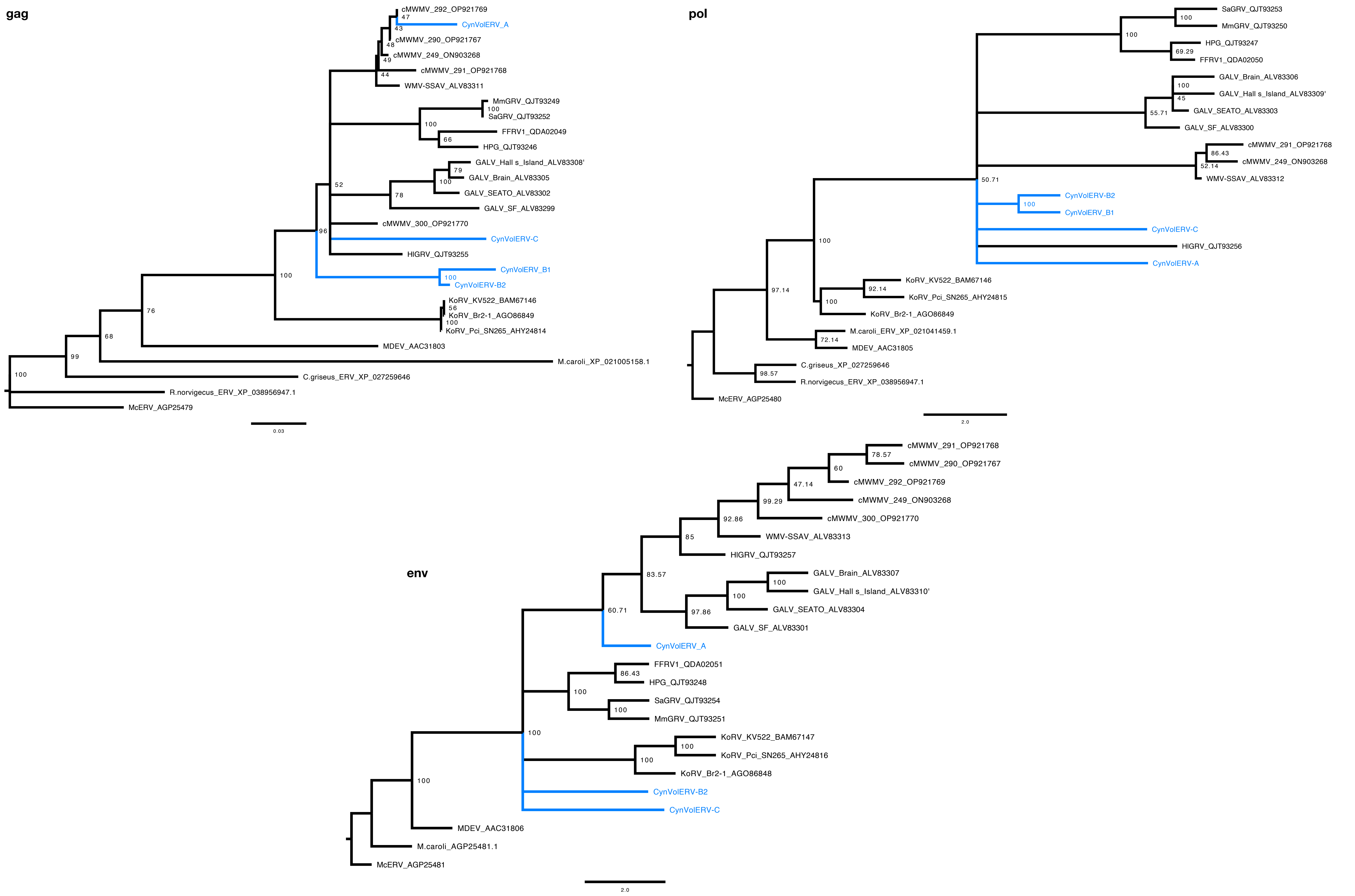

Fig S4: Phylogenetic trees generated with RAxML for proviral proteins, as predicted from each CynVolERV subgroup's consensus sequence by Retrotector. McERV sequence was used as an outgroup.

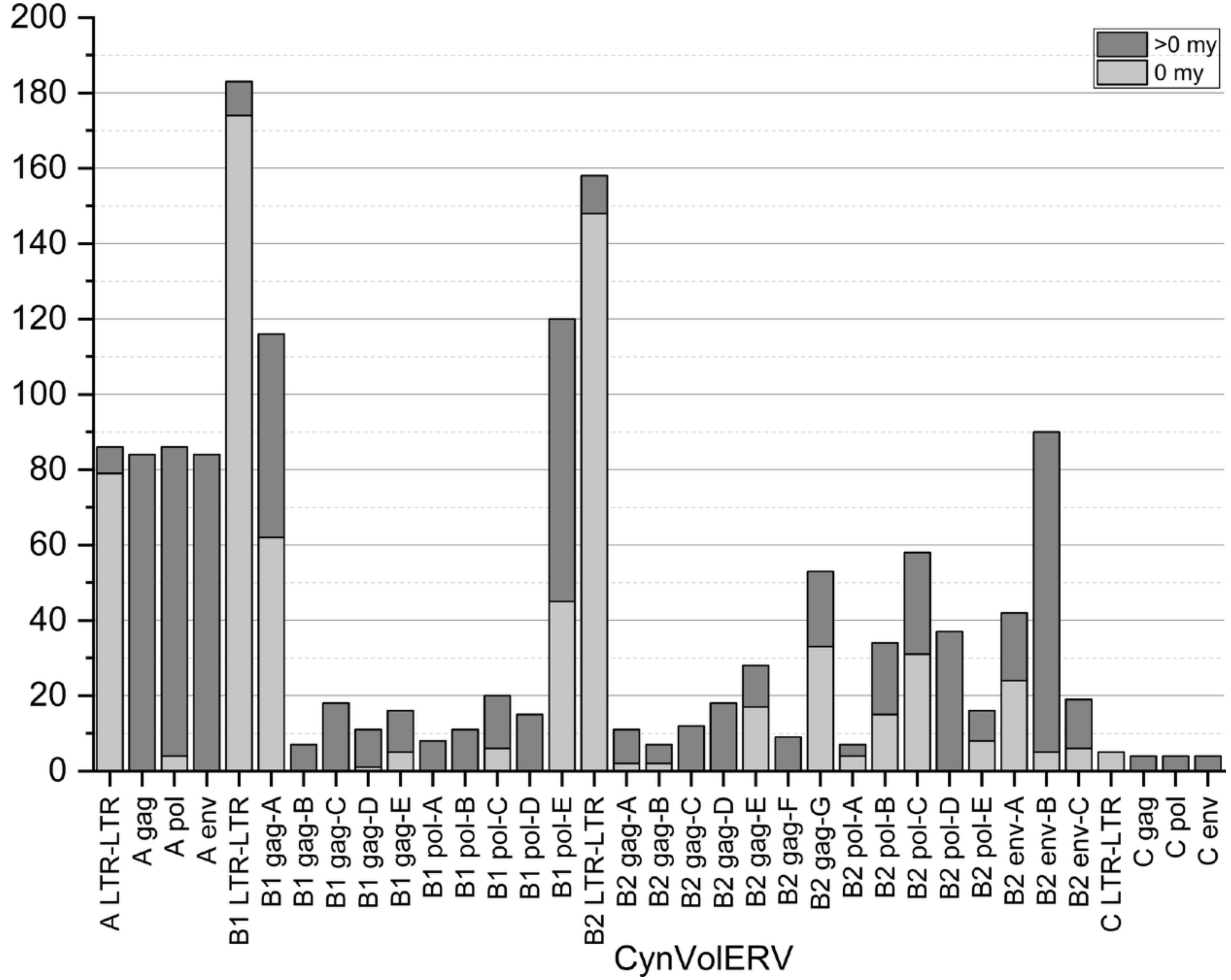

Fig S5: Stacked bar chart that illustrates zero vs. non-zero million years provirus age estimations
